# Supplementary figures and images for: Exercise Affects Mucosa-Associated Microbiota and Colonic Tumor Formation Induced by Azoxymethane in High-Fat-Diet-Induced Obese Mice
Source: Microorganisms. 2024 May 9;12(5):957. doi: 10.3390/microorganisms12050957 (PMC11124473; doi:10.3390/microorganisms12050957)

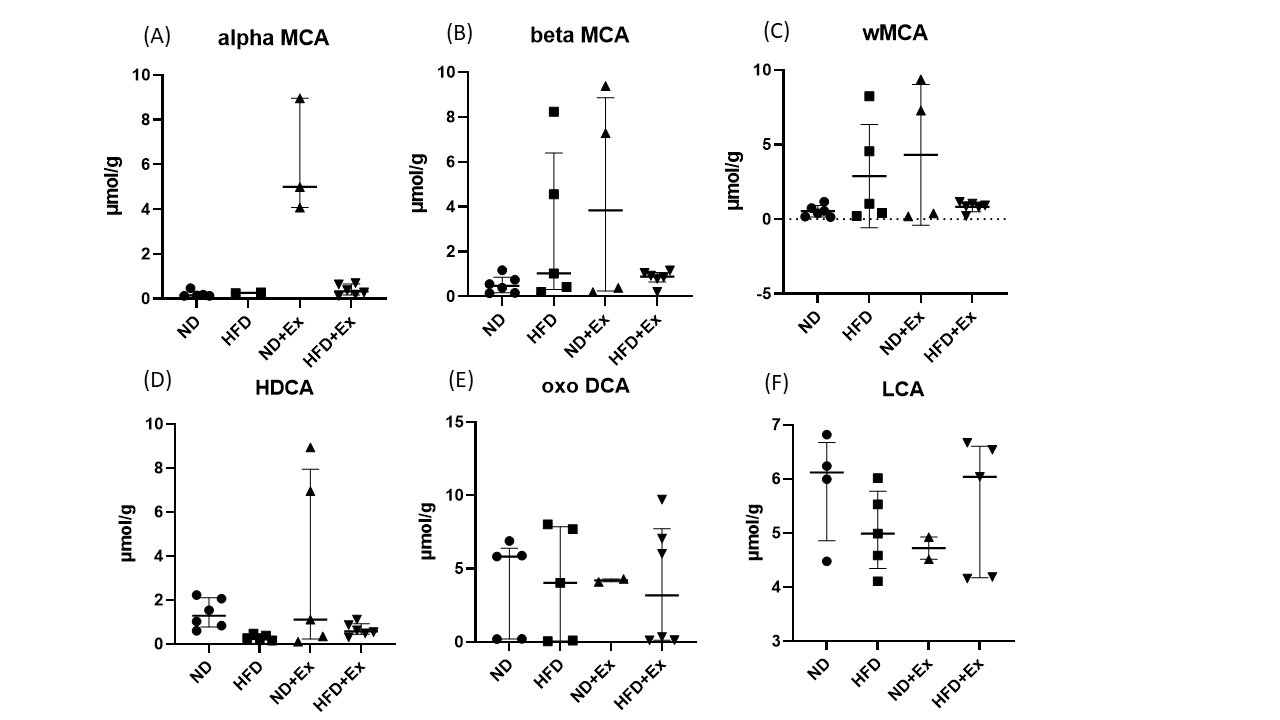

Supplement: Supplementary file 1 [file microorganisms-12-00957-s001.zip › microorganisms-2954048-supplementary/supplement figures/Supplement Figure S1A-F.tif]

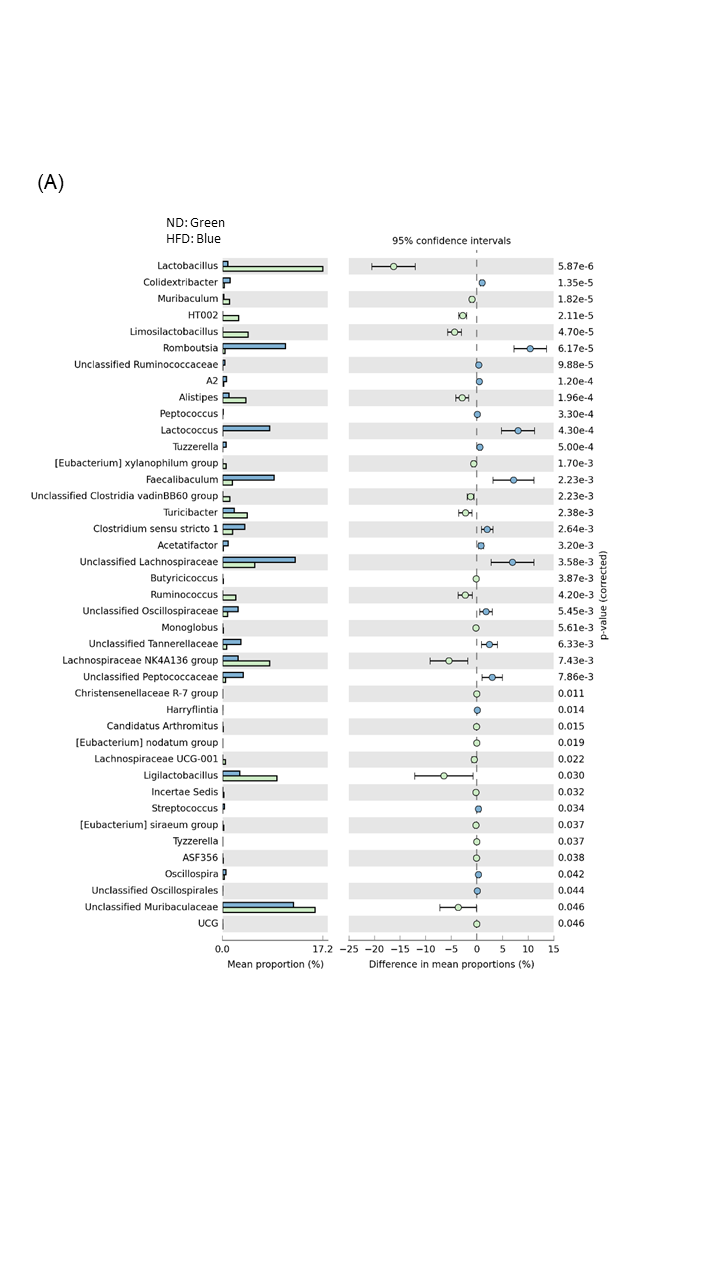

Supplement: Supplementary file 1 [file microorganisms-12-00957-s001.zip › microorganisms-2954048-supplementary/supplement figures/Supplement Figure S2A.tif]

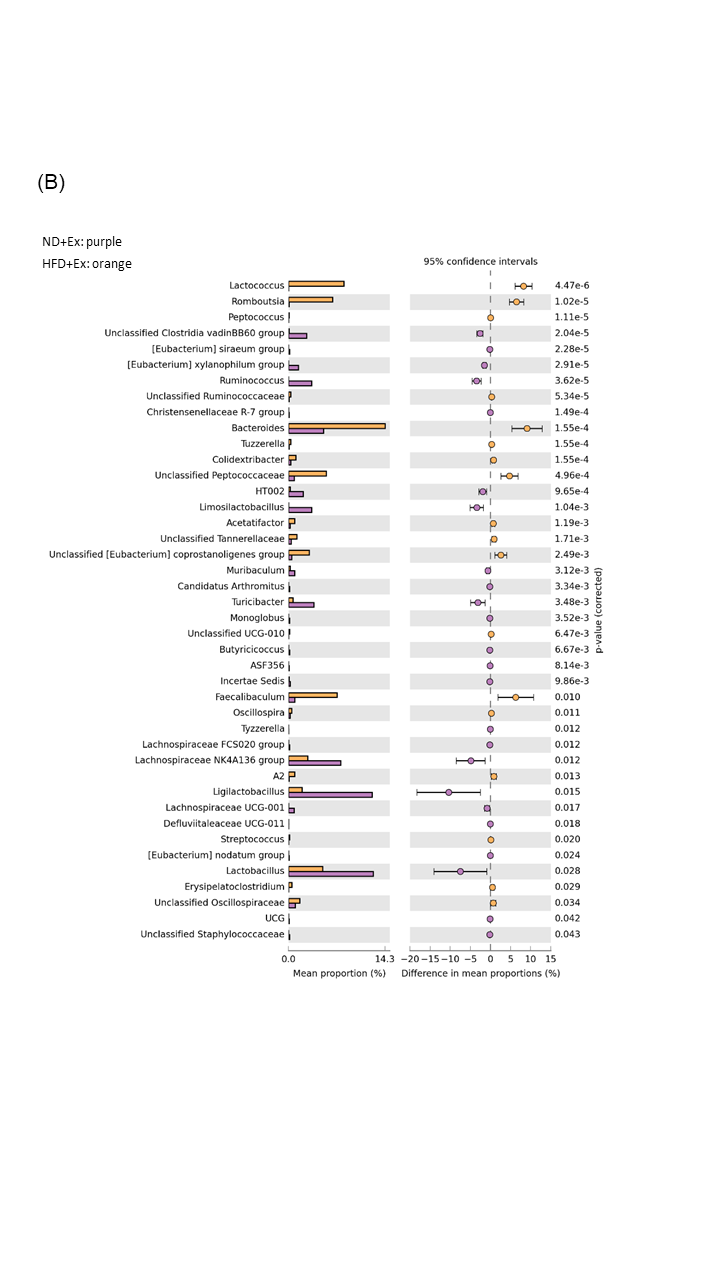

Supplement: Supplementary file 1 [file microorganisms-12-00957-s001.zip › microorganisms-2954048-supplementary/supplement figures/Supplement Figure S2B.tif]

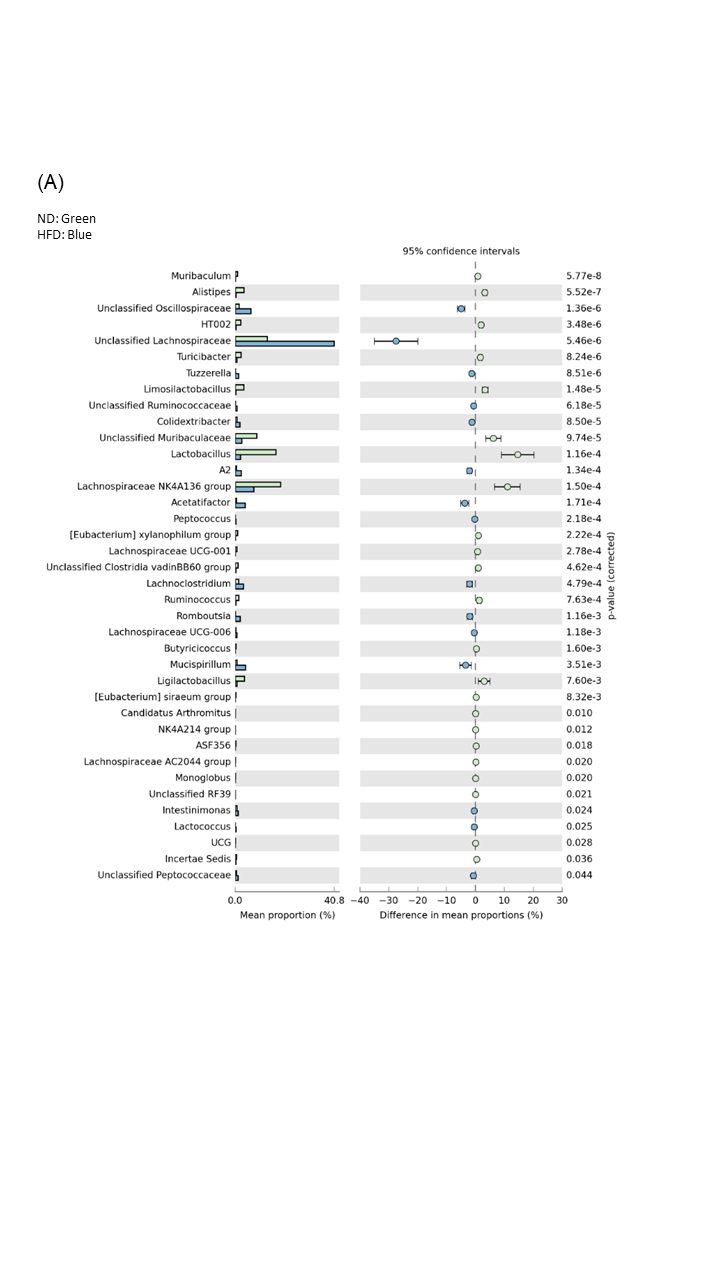

Supplement: Supplementary file 1 [file microorganisms-12-00957-s001.zip › microorganisms-2954048-supplementary/supplement figures/Supplement Figure S3A.tif]

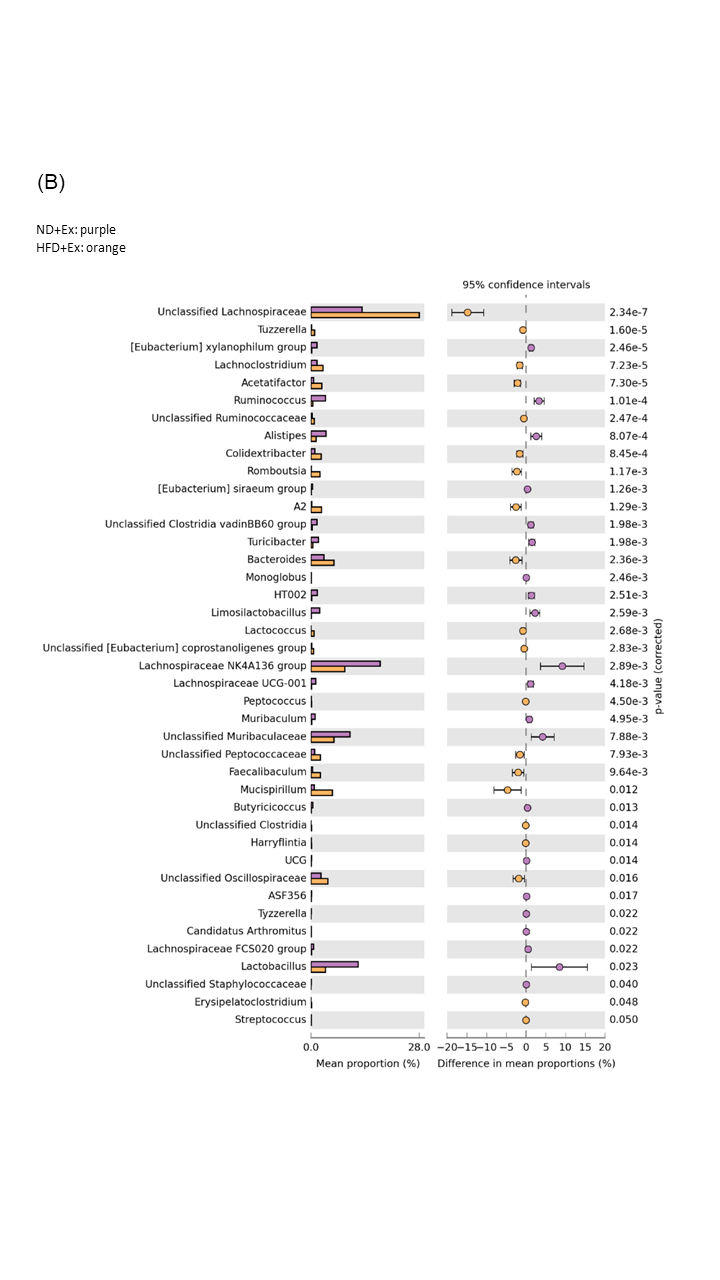

Supplement: Supplementary file 1 [file microorganisms-12-00957-s001.zip › microorganisms-2954048-supplementary/supplement figures/Supplement Figure S3B.tif]
